# Three QTL in the honey bee *Apis mellifera L.* suppress reproduction of the parasitic mite *Varroa destructor*

**DOI:** 10.1002/ece3.17

**Published:** 2011-12

**Authors:** Dieter Behrens, Qiang Huang, Cornelia Geßner, Peter Rosenkranz, Eva Frey, Barbara Locke, Robin F A Moritz, F B Kraus

**Affiliations:** 1 Institute of Biology, Martin-Luther-University Halle-WittenbergHoher Weg 4, 06099 Halle (Saale), Germany; 2 Honeybee Research Institute, Jiangxi Agricultural UniversityNanchang 330045, China; 3 Department of Anatomy and Structural Biology, University of Otago270 Great King Street, 9016 Dunedin, New Zealand; 4 Apicultural State Institute, University of HohenheimAugust-von-Hartmannstraße 13, 70599 Stuttgart, Germany; 5 Department of Ecology, Swedish University of Agricultural SciencesUlls Väg 16, 750–07 Uppsala, Sweden; 6 Department of Zoology and Entomology, University of PretoriaPretoria, South Africa

**Keywords:** Disease resistance, drones, microsatellites, quantitative trait loci

## Abstract

*Varroa destructor* is a highly virulent ectoparasitic mite of the honey bee *Apis mellifera* and a major cause of colony losses for global apiculture. Typically, chemical treatment is essential to control the parasite population in the honey bee colony. Nevertheless a few honey bee populations survive mite infestation without any treatment. We used one such *Varroa* mite tolerant honey bee lineage from the island of Gotland, Sweden, to identify quantitative trait loci (QTL) controlling reduced mite reproduction. We crossed a queen from this tolerant population with drones from susceptible colonies to rear hybrid queens. Two hybrid queens were used to produce a mapping population of haploid drones. We discriminated drone pupae with and without mite reproduction, and screened the genome for potential QTL using a total of 216 heterozygous microsatellite markers in a bulk segregant analysis. Subsequently, we fine mapped three candidate target regions on chromosomes 4, 7, and 9. Although the individual effect of these three QTL was found to be relatively small, the set of all three had significant impact on suppression of *V. destructor* reproduction by epistasis. Although it is in principle possible to use these loci for marker-assisted selection, the strong epistatic effects between the three loci complicate selective breeding programs with the Gotland *Varroa* tolerant honey bee stock.

## Introduction

The parasitic mite *Varroa destructor* is the most dangerous parasite of the western honey bee *Apis mellifera* (Rosenkranz et al. 2010). By feeding on the hemolymph of developing and adult bees, the mite damages the bees physically and physiologically. The most devastating effects of the mite, however, are caused by its ability to vector several highly pathogenic honey bee viruses, dramatically increasing viral disease in the colony and often leading to colony death (Böcking and Genersch 2008). So far more than 18 honey bee viruses have been described and many are associated with *Varroa* mite infestation, most notably deformed wing virus (Chen and Siede 2007; Ribière et al. 2008).

The problem arose four decades ago after the mite's transition from its original host, the eastern honey bee *A. cerana* (Oldroyd 1999). The mite spread across the globe within few decades and today only Australia (Oldroyd 1999; Anderson and Trueman 2000; Rosenkranz et al. 2010), northern Sweden and Norway (SJVFS 2010), some extremely isolated populations on islands (e.g. Ile d'Ouessant: Tentcheva et al. 2004), and remote oases in deserts (Shaibi et al. 2010) have managed to remain free of *Varroa* infestations.

With the exceptions of Africanized and African bee races, apiculture with the western honey bee is nearly impossible unless regular mite control treatments (usually chemical acaricides) are used to control the parasite population (Rosenkranz et al. 2010). In temperate climates, a colony, once it is infested with *V. destructor*, will collapse without mite control treatment within 2–3 years (Rosenkranz et al. 2010; Böcking and Genersch 2008). In the past decades, several chemicals have been used to control *V. destructor* infestations, but unfortunately the mite rapidly evolved resistance against these chemicals and their efficiency declined (Lodesani et al. 1995; Elzen and Westervelt 2004; Pettis 2004). In addition, control treatments often cause contamination of the apicultural products including acaricide residues in honey and pollen (Wallner 1999; Martel et al. 2007). It is therefore apparent that alternative strategies are needed to fight *V. destructor* that will neither facilitate resistance in the parasite populations nor contaminate bee products, thus ensuring both consumer health and customer trust in honey bee products.

In spite of the global Varroosis disaster, a few populations of European honey bees have been identified to survive infestations without any form of mite control treatment. These populations have not been managed by bee breeders but rather evolved tolerance through natural selection by mite infestation (De Jong and Soares 1997; Kefuss et al. 2004; Fries et al. 2006; Le Conte et al. 2007; Seeley 2007). *Varroa* tolerance may be based on very different traits, since the interaction between the mite and the host is very complex. A particularly well-studied behavioral trait that can lead to colony tolerance is the so-called hygienic behavior of the honey bee (Böcking and Spivak 1999). This trait is important for mite resistance of the eastern honey bee *A. cerana* (Peng et al. 1987) and has been in focus of various breeding programs in the western honey bee *A. mellifera* (Rinderer et al. 2010). Hygienic behavior has been shown to be controlled by quantitative trait loci (QTL) (Lapidge et al. 2002; Oxley et al. 2010) influencing the task thresholds for uncapping and removal of dead, diseased, or parasitized brood (Rothenbuhler 1964; Moritz 1988).

However, a more direct path toward mite resistance is the ability of the individual larva or pupa to prevent mite reproduction in the brood cell (Fries et al. 1994). The mite's reproduction is closely synchronized with that of the infested developing pupa, and different compounds of the larval cuticle are responsible for initiating egg laying by the mite (Garrido and Rosenkranz 2003, 2004).

After a decade of natural selection for survival without treatment, it has been demonstrated that mite reproductive success is reduced to about 50% in the honey bee population on the island of Gotland (Locke and Fries 2011). Cross-infestation experiments with the honey bee population on Gotland demonstrated that the observed mite tolerance in this population is a trait of the bees, and not one of the local mite population (Fries and Bommarco 2007). Mite infertility was one of the parameters influencing the reduced reproductive success of the mite in this population (Locke and Fries 2011) and is further a highly variable trait ranging between 5 and 20% in worker brood of European honey bees (Rosenkranz et al. 2010).

In this study, we aim to identify genomic regions, which influence the suppression of mite reproduction by honey bee larvae and pupae, to enable future marker-assisted breeding programs for *Varroa*-resistant honey bee stock. The availability of both the complete *A. mellifera* genome sequence (Weinstock et al. 2006) and the tolerant population on Gotland, provides an ideal setting to screen for QTL that interfere with *V. destructor* reproduction. Large sets of highly variable microsatellite markers covering the entire genome have been established (Solignac et al. 2003, 2007) and novel markers can be easily extracted from the genome sequence that can be used for high-density fine-scale mapping (Lattorff et al. 2007; Shaibi et al. 2008). In addition, because honey bees have a haplodiploid sex determination, the haploid drones provide an extremely simple and highly efficient model system for genetic studies (Moritz and Evans 2007; Moritz et al. 2010). Drones are also important for *Varroa* resistance from an epidemiological point of view, since *Varroa* mites preferentially reproduce in the drone brood of *A. mellifera* (Fuchs 1990). In the adapted host *A. cerana*, the mite reproduction is even completely restricted to the drone brood (Boot et al. 1999).

Here, we embark on using drones as a genetic model system to screen for QTL for suppression of *Varroa* mite reproduction. Because drones only have a mother queen and no father, it requires only a single generation to establish a mapping population of hundreds of individuals yielding an extremely powerful strategy for QTL identification.

## Methods

### Mapping population

The isolated honey bee population on the island of Gotland in Sweden has been under natural selection for mite tolerance for more than 10 years and has survived without any *Varroa* treatment (Fries et al. 2006; Locke and Fries 2011). Today, the Gotland population shows clear signs of tolerance toward *Varroa* mites and a significant reduction in the reproductive success of *Varroa* mites, whereas hygienic and grooming behavior of the bees is not increased (Locke and Fries 2011). A queen of pure Gotland origin was naturally mated to drones at the apiary of the University of Hohenheim, where the local population does not show any signs of *Varroa* tolerance or resistance and is considered to be genetically *Varroa* susceptible. Two hybrid F1 daughter queens of the Gotland queen (queen A and B in the following) were naturally mated and introduced into strong foster colonies with equally high *Varroa* infestation levels. Empty drone brood frames were added allowing the queens to produce a large drone mapping population.

### Phenotypic classification

Sealed drone brood cells were opened 15–18 days after egg laying and checked for *Varroa* infestation and reproduction of the mite. Pupae infested with only a single mite with no offspring were classified either as (1) resistant (*n* = 144) and those with at least three viable offspring mites as (2) susceptible (*n* = 635). Drone pupae with intermediate reproductive success of the mite (one or two offspring mites, *n* = 107) were not included in the mapping population. This selective DNA pooling approach (Darvasi and Soller 1994) with a focus on the extreme phenotypes allows for obtaining a clear-cut segregation of individuals and alleles. After the identification of the phenotype, all drone pupae were transferred into 90% ethanol and stored at –20°C until DNA extraction.

### DNA extraction and bulk segregant analysis (BSA)

Genomic DNA of all resistant (*n* = 144) and a subset of susceptible (*n* = 128) drone pupae was extracted individually from a leg, each following a modified Chelex extraction protocol (Biorad, Walsh et al. 1991). DNA concentrations were measured using the Nanodrop ND 1000 Spectrophotometer (peqlab, v 3.5.2) and equal amounts of DNA per individual were pooled according to the defined resistance phenotype from hybrid queen A (resistant, *n* = 32; susceptible, *n* = 48). We then genotyped these pools in a BSA with a total of 488 microsatellite markers distributed over all 16 chromosomes of the honey bee at 55°C following standard multiplex polymerase chain reaction (PCR) protocols (eight primer pairs per reaction; 35 cycles) (Michelmore et al. 1991; Solignac et al. 2003). Of these 488 microsatellite markers, 216 markers were heterozygous in mother queen A resulting in a resolution of one marker every 1 Mb or 19 cM on average. The mean distance between markers was 8.3 ± 0.3 cM, 78% of the genome was less than 5 cM and 96% less than 10 cM away from a heterozygous marker tested in the BSA. The marker coverage for each chromosome is illustrated in Figure S1. The obtained microsatellite fragments were analyzed with an automated DNA capillary sequencer (MegaBACE 1000) and scored with the MegaBACE Fragment Profiler Version 1.2.

For all markers, which were heterozygous in the mother queen, the fluorescence intensities (i.e., peak heights) of the two alleles (i.e., PCR products) were taken as an estimator for the allele frequencies in the DNA pools. In case of different allele frequencies between the pools (i.e., one allele predominant in one phenotypic pool, the alternative allele in the other), the ratios of the measured peak heights are expected to differ in the two PCR reactions accordingly. This difference was calculated as the sum of differences in the normalized fluorescence intensities of both alleles between the two phenotypic pools (see equation in Fig. S2) and used to select candidate regions for fine-mapping. All drones of the bulked DNA pools were then individually genotyped at these markers to confirm or reject a biased allele distribution in the phenotypic pools.

### Individual genotyping and QTL-mapping

Based on the results of the BSA, all individuals from hybrid queen A were individually genotyped at a total of 131 microsatellite markers to verify the QTL candidate regions. In a single marker analysis using the software Map manager QTX (Manly et al. 2001), we identified three candidate regions, where consecutive markers showed significant different frequencies in the two phenotypic pools (χ^2^-test, *P* < 0.05). We then genotyped 112 resistant and 80 susceptible individuals from hybrid sister queen B at 60 informative loci (Table S1) within these candidate regions identified in queen A to test whether the identified regions also caused a phenotypic segregation in the second mapping population. Both datasets were analyzed separately as well as pooled after reconstruction of the maternal F1 chromosomes from the haploid F2 drone offspring in both possible assignments (chromosome 1 in queen A assigned to chromosome 1 in queen B and to chromosome 2, respectively). If markers were homozygous in one of the two sister queens, these were treated as missing values in the respective part of the dataset. Pooling of datasets was done under the assumption that the resistance allele had gone to fixation in the selected and inbred Gotland population. Hence, the mother of the two sister queens is assumed to be homozygous for this allele and it must be shared by the two half-sister hybrid queens. To confirm the Gotland origin of the alleles in the resistant pool, we genotyped a pooled DNA sample of 74 drones caught at a drone congregation area on Gotland in 2007 on 40 markers in the candidate regions, and screened for common alleles to identify the maternal F1 chromosomes.

The genotypes and a binary trait value for each individual (0 for susceptible and 1 for resistant) were then entered into the software Map manager QTX (Manly et al. 2001) to calculate the suggestive and significant QTL thresholds separately for each candidate region (15,000 permutations) conducting single marker analysis and simple interval mapping. Marker positions were defined according to the genomic map Amel_4.5 (NCBI Map viewer, http://www.ncbi.nlm.nih.gov) and individuals were coded as double haploids. In addition, the R package R/qtl (Broman et al. 2003; R Development Core Team 2010) was used for simple interval mapping, to test for differences due to a software effect and for graphic display. Furthermore, the amount of phenotypic variance explained by each QTL separately in a single-locus model, as well as by significant epistatic interactions in a two-locus model was calculated using R/qtl. QTL regions were then screened for annotated genes in the honey bee genome database (NCBI Map Viewer; Amel_4.5).

## Results

### QTL candidate regions

**Figure 1:**
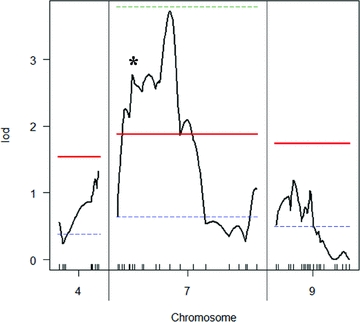
Candidate regions on chromosomes 4, 7, and 9 with their associated LOD scores from simple interval mapping of the pooled dataset using R/qtl. The vertical lines represent the QTL thresholds (blue dashed: suggestive, *P* < 0.63; red solid: significant, *P* < 0.05; green dashed: highly significant, *P* < 0.001) (15,000 permutations). The asterisk (*) on chromosome 7 indicates the approximate position of the “futsch” ortholog (GB11509).

Based on the BSA and the subsequent individual genotyping, three regions of interest, located on chromosomes 4, 7, and 9, were identified showing linkage of one or more markers in the single marker analysis to the defined trait value of host resistance. Both softwares used for simple interval mapping gave nearly identical results, indicating that the mapping results are robust, irrespective of the software applied. The results for the pooled dataset of the simple interval mapping in the three candidate regions using R/qtl are shown in Figure 1. Whereas the QTL regions on chromosome 4 (ranging from 2.1 to 4.3 Mb) and 9 (ranging from 1.0 to 3.5 Mb) were only suggestive in simple interval mapping and explained 5.3 and 3.7% of the phenotypic variance, respectively, the region on chromosome 7 (ranging from 3.6 to 8.5 Mb) significantly influenced the phenotype explaining 8.7% of the variance in a single-locus model. The majority of the designated “resistance” marker alleles in all three regions (62%, *n* = 80, Table S1) were also found in the drone sample from 2007 suggesting that our marker assignment corresponded to the alleles present in the Gotland population. This was further confirmed by the analysis of the alternative phase assignments that caused a complete loss of all QTL signals. A list of the 17 candidate genes located within a confidence interval around the highest LOD (Logarithm of the odds) score on chromosome 7 (LOD = 3.73 ± 1) is given in Table S2. The results from the simple interval mapping of the separate datasets for queen A and B are given in Figure S4.

### Interactions between QTL

**Figure 2:**
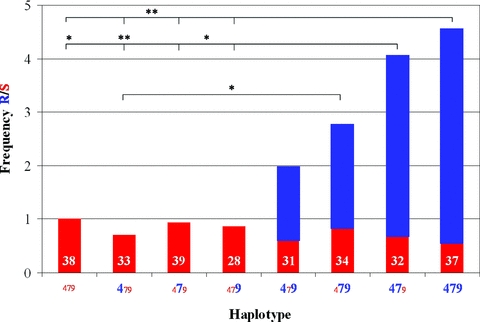
Frequency ratios between the number of resistant (R; *n* = 144) and susceptible (S; *n* = 128) individuals for all possible haplotypes at the three identified QTL, normalized for the frequency ratio found in the triple susceptible haplotype 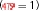. White numbers at the bottom of the bars indicate the number of individuals with the respective haplotype. Bold blue numbers represent the marker alleles associated with the resistant phenotype, whereas small red numbers denote those alleles associated with susceptible pupae. For example, “
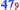
” represents individuals with the “resistance” marker alleles at the QTL on chromosome 4 and 7, but the alternative marker allele on chromosome 9 (*P < 0.05; **P < 0.01, two-tailed Fisher exact test). Blue bars illustrate the phenotypic effect of QTL interactions.

Using R/qtl and a two-locus model, we found a significant epistatic interaction of the QTL regions on chromosome 4 and 7 (markers UN071 and UN391). This interaction explained 3.5% of the phenotypic variance in the pooled dataset by interaction alone. In total, the two-locus model using these two markers explained 10.1% of the phenotypic variance in our dataset and was supported with a LOD score of 6.3. A heat map visualizing epistasis in the two-locus model is given in Figure S3. Figure 2 illustrates the epistatic effect on the phenotype and shows the frequency ratios between resistant and susceptible drone pupae within haplotype groups. Because we use haploid genotypes (and only because of this), it is possible to directly visualize and determine epistatic effects on the resistance trait. Drone pupae with a single resistance allele at one of the three loci did not significantly deviate from the triple susceptible haplotype. In contrast, the combination of the resistant alleles on chromosome 4 and 7 as well as the triple resistance haplotype have a more than fourfold increase in the likelihood to be resistant (two-tailed Fisher exact tests, Fig. 2). However, the interaction between the loci on chromosome 4 and 9 revealed no significant phenotypic effect and was not significant in the two-locus model. Since the individual single resistance alleles do not change the phenotype at all, but the combination of the three resistance alleles has a drastic effect, this is clear evidence of epistasis. Given the weak additive effects and the strength of epistasis, it is not surprising to see the LOD scores on chromosome 4 and 9 to be just suggestive in the individual mapping analyses.

## Discussion

The suppression of mite reproduction in the pupal stage of the host seems to be under significant control by three QTL located on chromosomes 4, 7, and 9. Although the individual Gotland alleles at each identified QTL had a low effect on pupal *Varroa* resistance (Fig. 2), and two QTL were not significantly supported by simple interval mapping (Fig. 1), these loci had nevertheless highly significant impact due to their epistatic interactions. The combination of the two Gotland alleles on chromosomes 4 and 7 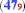 provided almost the same suppression of mite reproduction as the combination of all three resistance alleles 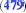 (Fig. 2). Hence, the combination of the two QTL on chromosome 4 and 7 are of prime interest when selecting for pupal *Varroa*-resistant phenotypes in the Gotland stock. Because of the complete lack of additive gene effects and the strong epistatic interaction, we recommend to select for this marker combination, although this complicates marker-assisted breeding attempts. The relevant alleles responsible for this particular tolerance trait in the Gotland population are listed in Table S1. Only because we used the simple genetic make-up of haploid drones, we have been able to detect the epistatic interaction as the main driver of suppression of *Varroa* mite reproduction, which may have remained undetected in a diploid study population.

### Detection limit

Before embarking on an in-depth discussion, the reader should be aware of the various general limitations and pitfalls of the QTL methodology (Slate 2005) including overestimation of QTL effects especially due to selective genotyping. The BSA in our study had an average resolution of about one heterozygous marker every 19 cM. Although major QTL are expected to produce large sweeps and should be detected even with a low density of markers, we may have missed minor QTL because of the selective genotyping approach (Darvasi and Soller 1994) and the high recombination rate of the honey bee genome. Therefore, like in any QTL study, there is a bias toward detection of major QTL versus minor QTL (Beavis 1994; Zeng 1994). In addition, the intrinsic inaccuracies of the standard bulk DNA samples analyses may not always reflect the actual genotype frequencies in the mapping populations (Michelmore et al. 1991). This can result in false positive or false negative signals and thus eventually to the nondetection of potential QTL. In spite of methodological imprecision, we are nevertheless confident to have identified three regions containing QTL involved in pupal *Varroa* resistance by suppressing mite reproduction. Clearly, we cannot exclude that additional loci that we have missed in the mapping procedure may also have been involved in the Varroosis-resistance phenotype.

### Candidate genes

The identification of functional genes in the identified target regions is definitely premature for the regions on chromosomes 4 and 9. Even, the significant QTL region on chromosome 7 includes 125 annotated genes. Nevertheless, it may be worthwhile to mention two of them. One is located directly at the LOD score peak on chromosome 7, which is the ortholog of the “foxo” gene (GB11764; see Table S2), a transcription factor of the insulin signaling pathway. This conserved pathway has been assigned to diverse functions in insect growth and body size development, immune response, longevity, nutrition, cell death, and energy metabolism (Nijhout 2003a, b; Wu and Brown 2006), for example, in *Drosophila* (Jünger et al. 2003), the *Culex* mosquito (Sim and Denlinger 2008) but also in humans (Willcox et al. 2008). The foxo gene therefore appears to be a suitable candidate gene to be involved in a trait expressed during pupal development in honey bees.

The second is the ortholog to the *Drosophila* gene “futsch” (GB11509, approximate position marked with “*” in Fig. 1). In a genome-wide expression study using microarrays, Navajas et al. (2008) found this gene to be significantly downregulated (0.86-fold) in a *Varroa*-tolerant honey bee line compared to a susceptible line. In *Drosophila*, this gene has been found to be downregulated in nonneuronal tissue during development (Hummel et al. 2000) and to be involved in phosphorylation and the induction of synaptic plasticity in neurons. Interestingly, most differentially expressed genes between the *Varroa*-tolerant and the susceptible line in the study of Navajas et al. (2008) were involved in neuronal development and sensitivity. Although these two may be promising candidate genes for a causative relationship, we cannot exclude this as pure coincidence or provide any biological explanation at the present stage.

### Impact on future breeding programs

The success of *Varroa* reproduction within the host brood cells is a crucial factor for a balanced host–parasite relationship. The most striking example is the original host, *A. cerana*, where mite reproduction is restricted to the drone brood (Boot et al. 1999) and where reproductive barriers exist between different haplotypes of the host and the parasite (Navajas et al. 2010). Suppression of reproductive success of *Varroa* females is considered as important tolerance factor in Africanized honey bees (Rosenkranz 1999) and has also been shown to be present in the European population from Gotland used in this study (Locke and Fries 2011). This suggests that selection of this trait can be achieved within the genomic architecture of the honey bee. A further advantage of the use of this trait in selection programs is that the effect on the phenotype can directly be controlled by analyzing the *Varroa* mite's reproductive success in the honey bee brood. It may therefore be highly rewarding to select for this trait in breeding programs for *Varroa* resistance. Because few genes can have major effects on this trait and individual genomes can be easily screened, marker-assisted selection (MAS) will facilitate breeding efforts more easily than for other traits that rely on complex behaviors of diploid workers (e.g., hygienic behavior). We strongly recommend taking advantage of haploid drones in mapping studies and suggest using them as a routine tool for implementing MAS in breeding programs of the honey bee. If we had not used haploid drones in this study but diploid workers instead, we very likely would have missed the significance of the strong epistatic interactions that drive the phenotype for *Varroa* resistance.

## Supporting Information

**Figure S1** Marker coverage as the proportion (%) ofthe *Apis mellifera* genome closer than 5, 10, 20,and 25 cM, respectively, to a heterozygous marker used in the BSAfor each chromosome and for the entire genome (Σ).

**Figure S2** Equation used for selection of candidate regions for fine-mapping.

**Figure S3** Heat map for the probability of epistatic interactions between two markers in our candidate regions for the pooled dataset.

**Figure S4** Candidate regions on chromosomes 4, 7, and 9with their associated LOD scores from simple interval mapping usingR/qtl for queen A (**A**) and queen B (**B**).

**Table S1.** Markers used for fine mapping.

**Table S2.** Annotated genes in genomic region linked to thehighest LOD score (LOD = 3.73 ± 1) on chromosome 7.
