# Supplementary figures and images for: Three QTL in the honey bee Apis mellifera L. suppress reproduction of the parasitic mite Varroa destructor
Source: Ecol Evol. 2011 Dec;1(4):451–8. doi: 10.1002/ece3.17 (PMC3287329; doi:10.1002/ece3.17)

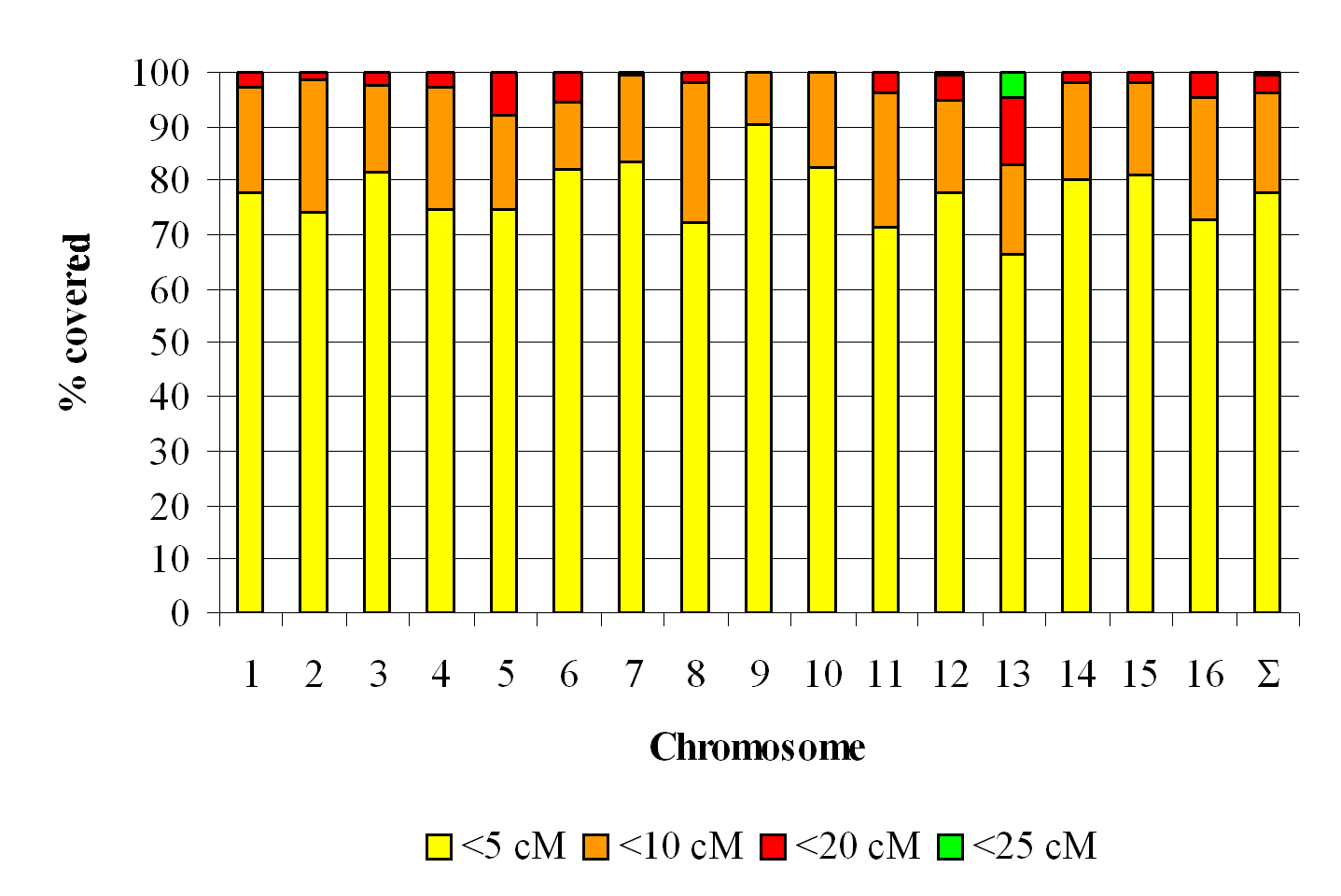

Supplement: Supplementary file 1 [file ece30001-0451-SD1.tif]

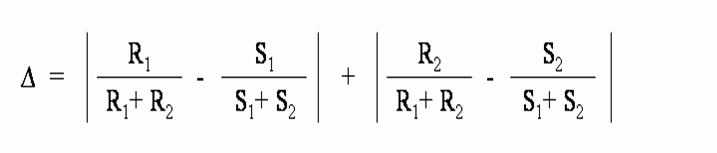

Supplement: Supplementary file 2 [file ece30001-0451-SD2.tif]

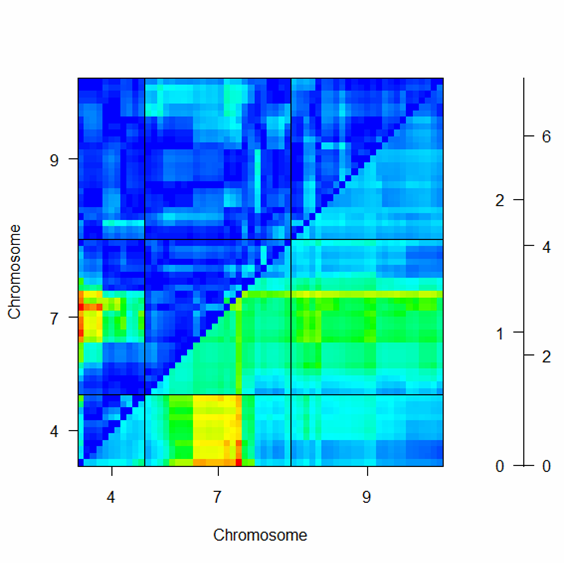

Supplement: Supplementary file 3 [file ece30001-0451-SD3.tif]

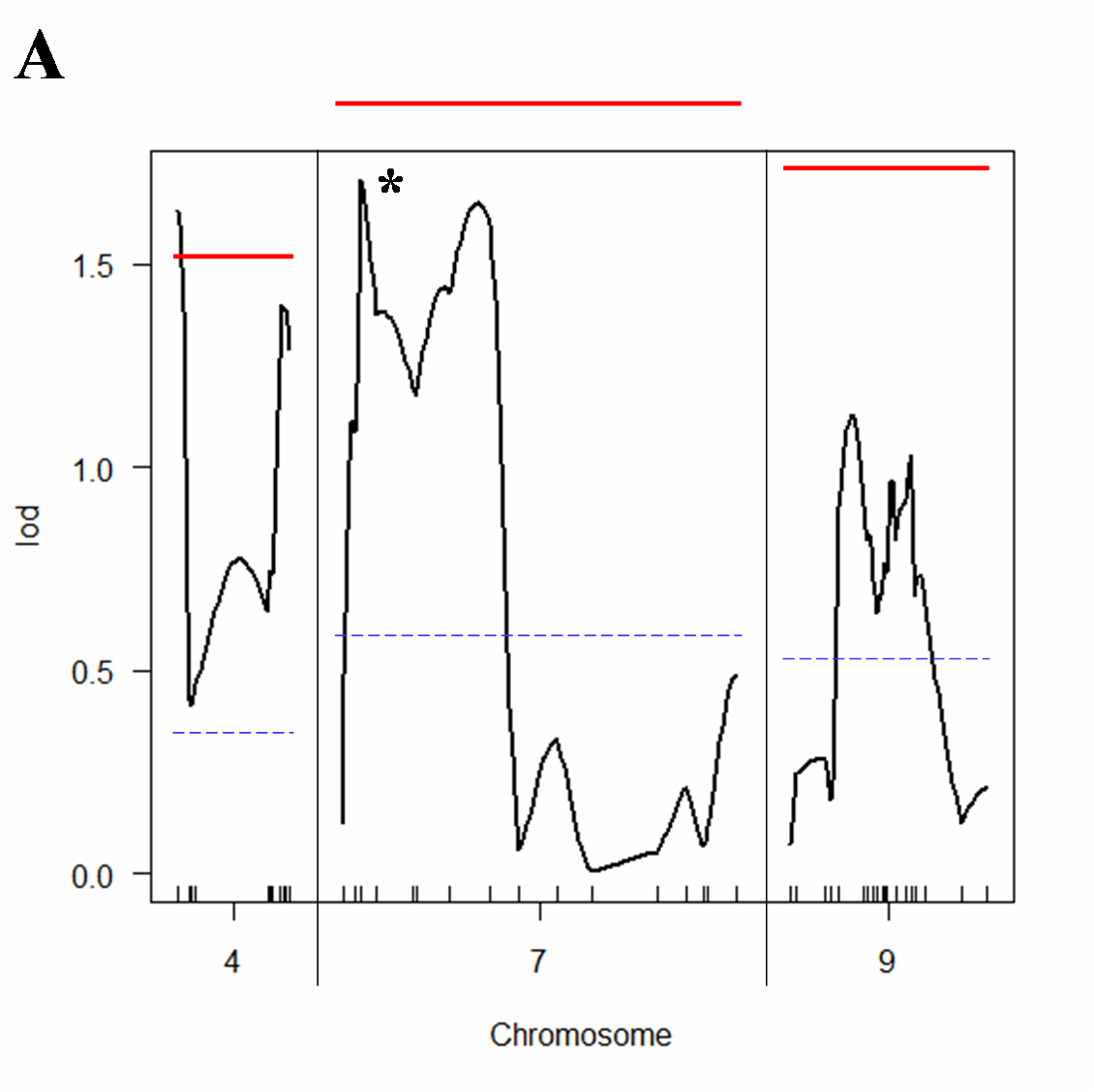

Supplement: Supplementary file 4 [file ece30001-0451-SD4.tif]

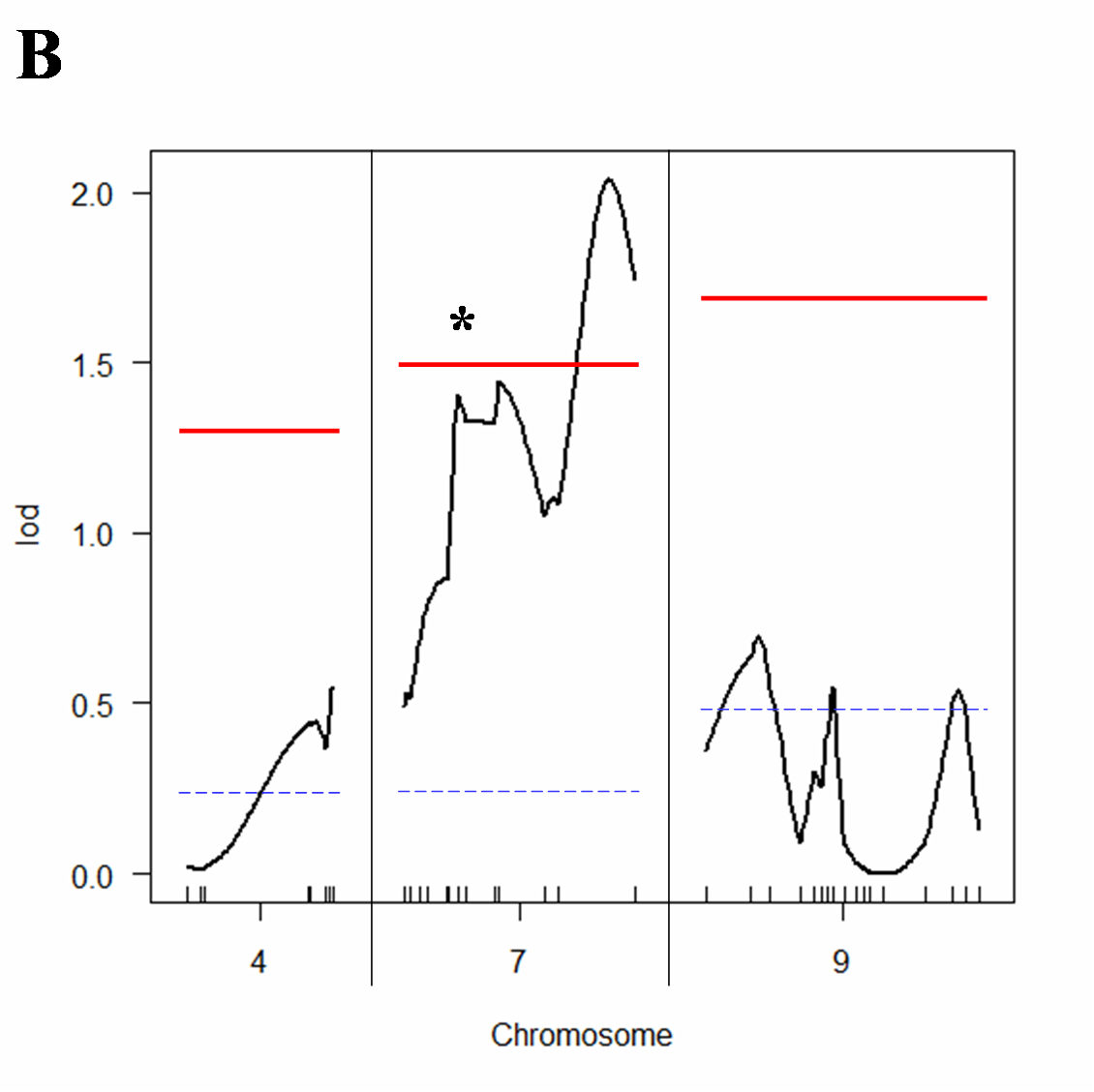

Supplement: Supplementary file 5 [file ece30001-0451-SD5.tif]
