# Supplementary material for: Three QTL in the honey bee Apis mellifera L. suppress reproduction of the parasitic mite Varroa destructor
Source: Ecol Evol. 2011 Dec;1(4):451–8. doi: 10.1002/ece3.17 (PMC3287329; doi:10.1002/ece3.17)
Supplement: Supplementary file 6 [file ece30001-0451-SD6.doc]

**Fig. 1** Candidate regions on chromosomes 4, 7 and 9 with their associated LOD scores from simple interval mapping of the pooled data set using R/qtl. The vertical lines represent the QTL thresholds (blue dashed: suggestive, P < 0.63; red solid: significant, P < 0.05; green dashed: highly significant, P < 0.001) (15.000 permutations). The asterisk (*) on chromosome 7 indicates the approximate position of the “futsch” ortholog (GB11509).

**Fig. 2** Frequency ratios between the number of resistant (R; n = 144) and susceptible (S; n = 128) individuals for all possible haplotypes at the three identified QTL, normalized for the frequency ratio found in the triple susceptible haplotype (479 = 1). White numbers at the bottom of the bars indicate the number of individuals with the respective haplotype. Bold blue numbers represent the marker alleles associated with the resistant phenotype, whereas small red numbers denote those alleles associated with susceptible pupae. E.g. “**47**9” represents individuals with the “resistance” marker alleles at the QTL on chromosome 4 and 7, but the alternative marker allele on chromosome 9 (*: P < 0.05; **: P < 0.01, two-tailed Fisher exact test). Blue bars illustrate the phenotypic effect of QTL interactions.

**Fig. S1** Marker coverage as the proportion [%] of the *A. mellifera* genome closer than 5, 10, 20 and 25 cM respectively to a heterozygous marker used in the BSA for each chromosome and for the entire genome (Σ).

**Fig. S2** Equation used for selection of candidate regions for finemapping. R_1_ and R_2_ are the peak heights (i.e. fluorescence intensities) of the alternative alleles 1 and 2 in the resistant pool, S_1_ and S_2_ are the peak heights in the susceptible pool.

**Fig. S3** Heat map for the probability of epistatic interactions between two markers in our candidate regions for the pooled data set. Top left: pairwise LOD scores for epistatic interaction. Bottom right: joint (additive and epistatic) LOD scores. The color ranges are scaled on the right side for the top left part (left scale) and the bottom right part (right scale).

**Fig. S4** Candidate regions on chromosomes 4, 7 and 9 with their associated LOD scores from simple interval mapping using R/qtl for queen A (**A**) and queen B (**B**). The vertical lines represent the QTL thresholds (blue dashed: suggestive, P < 0.63; red solid: significant, P < 0.05) (15.000 permutations). The asterisk (*) on chromosome 7 indicates the approximate position of the “futsch” ortholog (GB11509).

**Table S1** Markers used for fine mapping. Name, physical position in kilo base pairs [Kb], the resistant marker allele sizes in both hybrid queens in base pairs [bp], and the primer sequences are given for each marker. Markers named “HQ…” were newly developed for this study. All others are taken from the NCBI Amel_4.0 database. Bold marker names indicate the markers with the highest LOD score. Blue allele sizes are Gotland alleles confirmed by the bulked drone sample caught on Gotland in 2007.

|  |  |  | **Queen A** | **Queen B** |  |  |
| --- | --- | --- | --- | --- | --- | --- |
| **Chrom.** | **Marker** | **Kb** | **allele [bp]** | **allele [bp]** | **F Primer** | **R Primer** |
| **4** | UN071 | 1.542 | 148 | 147 | AATCTTGTAACGCAACTATCATTCC | AGCTGGAGGAATTAAGTGGTTATC |
|  | UN069 | 1.795 | 99 | 98 | AACGGTCTCGCTTACCTGTCT | AGCGCTGATGATGGAGGAC |
|  | K0430B | 1.870 | 134 | 134 | CCACTCAGCGAGCGGATA | CACGATACCTCACGCTCGA |
|  | AC108 | 1.976 | 146 | 143 | GGCATTCGGCGGAGATTC | CCATTTCGAGGATGAGTTCTCG |
|  | HQ4408 | 3.757 | 214 | 214 | TCTTGTCTGTGGACAACGGACGC | ATCAGATGCTGACGGTGAACCCAAG |
|  | HQ4406 | 3.814 | 289 | 289 | AGAACCGGAAGTGTAGAACGGGAAC | GCGAGTATGTAGAGCGAGTGTGAGAGT |
|  | HQ4405 | 3.860 | 169 | 171 | TGAAAACTCTCTGGTGGTCGTGCAT | TTCTCTCTCGCAAGCTCATCCAGTC |
|  | AT047 | 4.050 | 134 | 130 | GGTAAACACGTGCCACCATC | TGGAATGTATCTCGACGATTCTC |
|  | HQ4402 | 4.160 | 224 | 238 | TCCATCATACGTGACCGAGGAACAC | TTAACTTAATCGGCATTGCGGGCTG |
|  | HQ4401 | 4.203 | 267 | 251 | ATGCGATGCATACGACTCAGCATGT | TGGGATTGAACCCAAAGCCTTTCTCT |
|  | **K0429** | 4.272 | 156 | 152 | CTCTTGATTGAAAGAAACTCGCC | AATAACGGTGTCATGCCGC |
| **7** | K0720 | 3.298 | 191 | 202 | TTCTCGGCTCGAAATCGTG | GTGGTTCAGAGCGCCAGAG |
|  | HQ7593 | 3.311 | 189 | 198 | CTTCTGTTGTCTCGCGACCGTTGA | CCCCTGGCTAGGAAAAGTTCGTTCC |
|  | HQ7595 | 3.431 | -- | 219 | CGTTCGCGTTAGGAAAAATGATCCCG | CTGTCTGCTCTTCTTTCGAGGGGGA |
|  | K0718 | 3.595 | 182 | 185 | GCCTGGATTAAGATTCTCACGA | CGTGGATTAATCCGAGCG |
|  | UN378 | 3.751 | 107 | 95 | GAAGACGGCTCTCGACCG | CCAGGTGCATGTCTCGCTC |
|  | SV200 | 4.103 | 146 | 148 | AATCGTGGGTTGGAAGAACAG | CAGTCCCTTCGAAACCGTC |
|  | HQ7622 | 4.135 | 173 | 151 | TCGATGCGGCGAAAATCTCCTCTTT | TGCATGGAGTTCGACTTGCAAAGGT |
|  | HQ7623 | 4.140 | -- | 204 | AGTTTATTCCTGACCCGGTTTCGCT | CGCCGTTCCCCATTCATATTTGACG |
|  | HQ7631 | 4.327 | -- | 230 | CTCACACTTAAACCACGTGCACACA | GGGGTTCGTACAGGTGTTGAGGTTC |
|  | UN290b | 4.478 | 204 | 204 | ATTCGTTCATTCGATGCTTCC | GTATTCGTCATCCGTAGCAAGAA |
|  | HQ7671 | 5.000 | 195 | 196 | TAAGAGAAAGCTCGAAAGTGGCGGG | ATTCTAATCGGAGCAACCGCGTCAA |
|  | A107 | 5.000 | 160 | 162 | CCGTGGGAGGTTTATTGTCG | GGTTCGTAACGGATGACACC |
|  | HQ7691 | 5.088 | 235 | 225 | CGGGAGCAACAACGAAGAAGGAACT | CGCGGTGCCCCTTTTTACAGTAATC |
|  | AT133 | 5.940 | 102 | 104 | CGCTTTAGCCGTCCCA | GGATTGTAGCGAGAGGATGA |
|  | UN334d | 6.214 | 126 | 147 | TTACGATTGGGAACCGGG | CCCAAACAATCGGAGGCA |
|  | **UN391** | 6.922 | 129 | 121 | GTCGAATGGCTAGAGACAAAGATG | CCGAATTGTCGATATCGCAT |
|  | K0715 | 7.633 | 309 | 309 | ACAGAAGCTCGAACACGATACC | AGTGGTCGATAACGCCGAG |
|  | K0706 | 8.562 | 161 | 160 | TCAGAGTGTCGTAACGAACAACC | TACGTCGCGCAGGTGTTC |
|  | SV159 | 9.421 | 162 | 161 | AACGGCTACACGAGTACGGTAT | CGATTCAAATTGAATTTATCATTCG |
|  | SV158 | 11.028 | 260 | 244 | AATTGACTATAATTGACATATGAGTGACG | CTCCAAACATAGACTGGTTGCG |
|  | SV272 | 11.738 | 178 | 176 | ATCAAAGACATCGGAGAACAAAGC | CCGCTCTAATTTCCCTAGATCTATCC |
|  | K0758B | 12.186 | 98 | 104 | TTACTCGTTGTCGCTTATTTTCAA | AATTCGCCAATCCCGTTG |
|  | K0757 | 12.284 | 129 | 126 | GCCATTGCCGTGGATTTAC | CCGTGAGAATAATCCGTGATTC |
|  | AC174 | 12.995 | 104 | -- | TCGTCGTAGCAGCAACGG | CGTAACGTAAGCGCGTCG |
| **9** | UN336 | 972 | 206 | 206 | TGACTATCTCCCTCTTTATGTCTTCG | TAGTTTTACGGATCAAAATCACTCAA |
|  | BI288 | 1.126 | 136 | -- | ATACGCGCGCTCACACAC | AGGATGCTATCGCACGGC |
|  | AT165 | 1.816 | 283 | 287 | GCGACCACGTTTAACAGGAC | ACCAGTGAATTTGTTCATCGC |
|  | UN085 | 1.970 | 177 | 177 | CCGCTACAAGATAACCGCATTAC | CGATAACACGATTCCTCGTGG |
|  | **UN086** | 2.167 | 167 | 163 | CCAATGAATGGGTAAATCTAGCTC | TGTAGAATTCCATTGGCAACG |
|  | HQ9491 | 2.732 | 172 | 174 | GTACAATGATCTCTCGGTGAGGCGG | TAGACGATAAGCAGCCTGAACCACG |
|  | K0633 | 2.754 | 133 | 135 | GAAAGTCCTTGCCATCGACC | GTCTTAGGTCGAGGCGAAGTC |
|  | K0632B | 2.867 | 192 | 188 | TGGAGAAGTGACTTCTAAACTGCA | TCGTGGATAAATTTATGGATGAATC |
|  | HQ9484 | 2.994 | 293 | 293 | CGCAACGACGTTCTACGAGGATG | CCTCGTTACGGAGGTAGCTGAAAGAT |
|  | HQ9483 | 3.116 | 185 | 199 | ACCGGTCCCTCAAACTTTCATACTGTG | CAACGGTGACGTCGGATACTTCACT |
|  | SV179 | 3.224 | -- | 182 | ACGAATACACGCGTAGACGC | CCGCGTTACTTGTCCATGTG |
|  | HQ9482 | 3.239 | 295 | 299 | TAGCGCGACATTCCAACGTAGGTG | TGGCAGAGGCTTAATCCTGAACAACT |
|  | UN210 | 3.307 | 111 | 109 | TGTCCGCTCGTCACATGC | CGGATCTGCGTCATCCCT |
|  | HQ9481 | 3.337 | 199 | 174 | CGTGGAACATGGGACAGTGTTACGA | AACAATGTTACAGCGACCGCAAACC |
|  | AP005 | 3.555 | 140 | 139 | TCGAGAATTTCAGTCATCGC | CATTTAATCGACAATTCCGC |
|  | A055 | 3.791 | 194 | 194 | GATCTTCGCGCGTTCCAC | TTAATGAAATCACCCTCCGG |
|  | K0919 | 3.932 | 190 | 190 | TTCCGACCCCTGATCTCG | ATTCTGAAATGGCCGATGG |
|  | BI372 | 4.035 | 250 | 252 | GAGCCAAACCGTTAACATTGTG | CAATATATTAATTAATATACTTAATCATCTGCG |
|  | K0917 | 4.269 | 155 | 155 | CCGTTAAATACTCGCGAAACG | GTGAACGCGTTTTACGACGT |
|  | HQ5615 | 5.063 | 183 | 192 | GCATGCGCAAATGCGTGTCG | CCCTCTCTCGCGCTCTCGTT |
|  | UNDW13 | 5.193 | 194 | 192 | ACATCGAGTAGCTAGAGCCGG | TTACTAGTGTAATGGCAAGACCCTAA |
|  | HQ5712 | 5.581 | 242 | 232 | TCGAAACAACGCGAAGTGGACG | AGCGGCTCCCGACATACCAA |
|  | K0955B | 5.586 | 205 | 205 | AATGGCGAGTCATCGAGTGTA | ATCTCGAATCGATGAAGGAGG |
|  | UN006 | 5.815 | 222 | 222 | GGCGAACTGGGTCAATTAGAG | GATCCTATCTTCGTGTTCCGC |
|  | K0961 | 6.072 | 175 | 181 | ACTCGCCTCCAGGACACG | ACTCGTCCATGGAAACTCGAC |

**Table S2.** Annotated genes in genomic region linked to the highest LOD score (LOD = 3.73 ± 1) on chromosome 7. A consecutive number, the contig on the respective chromosome, the starting position in kilo base pairs [Kb], the official gene ID, a description of its presumed function, and the gene ID of the *Drosophila melanogaster* ortholog if available are given. Bold positions mark the location of the highest LOD score in simple interval mapping.

| **Nr.** | **Contig** | **Position [Kb]** | **ID** | **Description / Homologies** | **Fly ortholog** |
| --- | --- | --- | --- | --- | --- |
| **1** | 7.17 | 6.220 | GB18884 | nicotinic acetylcholine receptor alpha4 subunit | CG12414 |
| **2** | 7.17 | 6.363 | GB12612 | nicotinic acetylcholine receptor alpha4 subunit | CG12414 |
| **3** | 7.17 | 6.376 | GB13746 | Snrpc, U1 small nuclear ribonucleoprotein C | CG5454 |
| **4** | 7.17 | 6.378 | GB16050 | RNA degradation | CG9715 |
| **5** | 7.17 | 6.384 | GB16379 | CCR4-NOT transcription complex subunit 6-like | CG31137 |
| **6** | 7.18 | 6.575 | GB13854 | similar to Tankyrase-1 | CG33106 |
| **7** | 7.18 | 6.717 | GB14497 | CCR4-NOT transcription complex subunit 6-like | CG31137 |
| **8** | 7.18 | 6.724 | GB30367 | twin protein | CG31137 |
| **9** | 7.18 | 6.731 | GB16879 | mitochondrial ribosomal protein S24 | CG13608 |
| **10** | 7.18 | **6.752** | GB11764 | foxo, forkhead box protein O | CG3143 |
| **11** | 7.18 | **6.947** | GB13873 | foxo, forkhead box protein O | CG3143 |
| **12** | 7.19 | 7.135 | GB14642 | zormin protein | CG1915 |
| **13** | 7.19 | 7.258 | GB15230 | zormin protein | CG1915 |
| **14** | 7.19 | 7.328 | GB10033 |  |  |
| **15** | 7.19 | 7.341 | GB19142 |  |  |
| **16** | 7.19 | 7.354 | GB15635 |  |  |
| **17** | 7.19 | 7.368 | GB30232 |  |  |
